# Biomarkers in Laryngeal Squamous Cell Carcinoma: The Literature Review

**DOI:** 10.3390/cancers15205096

**Published:** 2023-10-22

**Authors:** Barbara Verro, Carmelo Saraniti, Daniela Carlisi, Carlos Chiesa-Estomba, Antonino Maniaci, Jerome R. Lechien, Miguel Mayo, Nicolas Fakhry, Marianna Lauricella

**Affiliations:** 1 Division of Otorhinolaryngology, Department of Biomedicine, Neuroscience and Advanced Diagnostic, University of Palermo, 90127 Palermo, Italy; carmelo.saraniti@unipa.it; 2 Section of Biochemistry, Department of Biomedicine, Neuroscience and Advanced Diagnostic, University of Palermo, 90127 Palermo, Italy; daniela.carlisi@unipa.it (D.C.); marianna.lauricella@unipa.it (M.L.); 3 Head and Neck Surgery Research Group of Young Otolaryngologists of International Federation of Otorhinolaryngological Societies (YO-IFOS), 75000 Paris, France; carlosmiguel.chiesaestomba@osakidetza.eus (C.C.-E.); antoninomaniaci@unikore.it (A.M.); jerome.lechien@umons.ac.be (J.R.L.); miguelmmy@gmail.com (M.M.); nicolas.fakhry@ap-hm.fr (N.F.); 4 Otorhinolaryngology-Head & Neck Surgery Department, Donostia University Hospital, Biodonostia Research Institute, Faculty of Medicine, Deusto University, 20014 Donostia, Spain; 5 Faculty of Medicine and Surgery, University of Enna “Kore”, 94100 Enna, Italy; 6 Division of Laryngology and Bronchoesophagology, Department of Otolaryngology-Head and Neck Surgery, Epicura Hospital, University of Mons, B7000 Mons, Belgium; 7 Department of Otorhinolaryngology—Head and Neck Surgery, Complexo Hospitalario Universitario A Coruña (CHUAC), 15006 A Coruña, Spain; 8 Department of Otorhinolaryngology—Head and Neck Surgery, Hospital San Rafael (HSR), 15006 A Coruña, Spain; 9 Department of Oto-Rhino-Laryngology Head and Neck Surgery, La Conception University Hospital, Assistance Publique—Hopitaux de Marseille, Aix Marseille University, 13005 Marseille, France

**Keywords:** larynx, laryngeal cancer, biomarkers, heat shock proteins, metallothioneins, nuclear factor erythroid 2-related factor 2, heme oxygenase, cyclooxygenase-2, micro ribonucleic acid

## Abstract

**Simple Summary:**

Laryngeal squamous cell carcinoma is a prevalent cancer associated with poor prognosis in advanced stages. Despite advancements in diagnostic tools (e.g., narrow-band imaging), there have been minimal improvements in therapeutic approaches. The potential new frontier lies in the realm of biomarkers. This review aims to outline the current understanding of biomarkers in laryngeal cancer. Specifically, it concentrates on potential biomarkers, including heat shock proteins, metallothioneins, nuclear factor erythroid 2-related factor 2, micro ribonucleic acids, heme oxygenase, and cyclooxygenase-2. This review provides a survey of the existing literature on their role in laryngeal cancer. It also underscores the scarcity of the literature on this subject, highlighting the significant role of biomarkers in formulating more precise therapeutic strategies for individual patients.

**Abstract:**

Laryngeal squamous cell carcinoma (LSCC) is the second most common cancer among head and neck cancers. Despite a lower incidence of laryngeal carcinoma, new diagnostic techniques, and more targeted therapies, the overall survival has not changed significantly in the last decades, leading to a negative prognosis in advanced stages. Recently, several studies have focused on the identification of biomarkers that may play a critical role in the pathogenesis of LSCC. Reviewing the literature on the main databases, this study aims to investigate the role of some biomarkers in LSCC that are correlated with oxidative stress and inflammation: heat shock proteins; metallothioneins; nuclear factor erythroid 2-related factor 2; heme oxygenase; cyclooxygenase-2; and micro ribonucleic acids. This review shows that biomarker expression depends on the type, grade of differentiation, stage, and site of carcinoma. In addition, the role of these biomarkers in LSCC is still little-known and little-studied. However, the study of biomarker expression and the detection of a possible correlation with patients’ epidemiological, clinicopathological, and therapeutics data may lead to better awareness and knowledge of the tumor, to the identification of the best therapeutic strategy, and the most proper follow-up protocol tailored for each patient. In conclusion, the achievement of these goals may improve the prognosis of LSCC patients.

## 1. Introduction

Carcinoma of the larynx is the second most common cancer among head and neck cancers, with 177,422 new cases per year and 94,771 deaths per year worldwide [1]. Laryngeal cancer mainly affects the adult population aged between 55 and 65 years old, but cases in young adults (under the age of 40) have also been described [2]. To date, men are five times more affected than women, although there is a progressive increase in incidence in women due to the increased spread of the smoking habit; smoking habit and alcohol abuse represent the leading and main risk factors [3]. Other independent risk factors are occupation-related toxic agents such as polycyclic aromatic hydrocarbons, asbestos, wood dust, cement dust, etc. [4]. Laryngopharyngeal reflux represents another chronic stress factor that could increase the risk of laryngeal cancer, especially bile acids [5]. Regarding histology, squamous cell carcinoma (SCC) accounts for about 95% of laryngeal tumors, with the prevalence of low and moderate grades of differentiation [6]. The glottis is the most commonly affected laryngeal region (about 65–70% of cases), followed by the supraglottis and subglottis [7]. Biopsy with histological examination of the sample continues to be the gold standard for diagnosis. There are different surgical and non-surgical strategies to manage laryngeal squamous cell carcinoma (LSCC) [8]. Depending on the tumor stage, surgical therapy may involve organ preservation approaches (transoral laser microsurgery or transoral robotic surgery), open partial laryngectomies, or total laryngectomy. Non-surgical treatment may involve exclusive radiation therapy (RT), concurrent chemoradiotherapy (CRT), and, in some cases, immunotherapy. The choice of therapy, surgical or non-surgical, depends not only on the features of carcinoma but also on the patient in terms of age, comorbidities, preference, and socio-family background. Despite the fact that policies against smoking and alcohol have led to a lower incidence of laryngeal carcinoma (which today is around 60%) and despite the introduction of more sensitive diagnostic techniques [9], as well as more targeted therapies, 5-year overall survival (OS) has not changed significantly in recent decades leading to a negative prognosis due to late diagnosis in advanced stages in about 60% of cases with a survival rate below 50% [8,10].

So, based on these assumptions, in recent years, new studies have investigated the detection of biomarkers in laryngeal tumor tissue that could help to better characterize the LSCC and could better define a target population to whom a specific therapy may be proposed (RT, CRT, immunotherapy) with greater success rate. For instance, to date, there is an ongoing clinical trial about the possible relationship between the expression level of Excision Repair Crossing Complementation group 1 (ERCC1) biomarker and CRT or chemotherapy alone in locally advanced head and neck squamous cell carcinoma [ClinicalTrials.gov ID NCT02128906]. Moreover, a recent review focused on the most studied biomarkers in LSCC that are molecules involved in apoptosis (e.g., Bcl-2), in the cell cycle (e.g., cyclin D1, ki-67), structural proteins (e.g., E-cadherin, CD44), tumor suppressor genes and oncogenes (p53). To date, Bcl-2′s role in LSCC is not clear: some studies did not find any correlation between its expression and LSCC, while other studies reported its overexpression in nodal metastasis, advanced stages, and RT-resistant LSCC. Cyclin D1 is involved in cell progression to the S phase; however, it can also act as an oncogene in several tumors, e.g., breast, esophageal, lung, and prostate cancers. In LSCC, upregulation of cyclin D1 is associated with nodal metastasis. Ki-67 is another protein involved in cell cycle progression that is overexpressed in advanced LSCC with a worse prognosis. Proteins involved in cell adhesion and migration, such as E-cadherin, play an important role in tumor progression and involvement of lymph nodes. That is why E-cadherin is downregulated in advanced and poorly differentiated LSCC with nodal metastases. P53 is a tumor suppressor gene that, if mutated (e.g., in case of loss of function), promotes tumorigenesis. Three studies found that its upregulation is related to a worse prognosis in LSCC [11,12,13,14,15]. Despite these being the most studied biomarkers, the role of these biomarkers in the pathogenesis of LSCC is still unclear, and their use in characterizing LSCC in terms of prognosis and therapeutic response has potential but is not yet a reality.

Notably, the main risk factors for laryngeal carcinomas (smoking habit, alcohol abuse, and polycyclic aromatic hydrocarbons) are known to induce cellular ROS production and oxidative stress [16,17,18], an event that is strictly correlated with tumorigenesis [19].

In this vein, we focused our analysis on factors (transcription factors, heat shock proteins, antioxidant enzymes) whose expression levels change under oxidative stress. In particular, this study aims to investigate protein chaperone biomarkers such as heat shock proteins (HSPs), antioxidant proteins such as metallothioneins (MTs), transcription factors such as nuclear factor erythroid 2-related factor 2 (Nrf2), enzymes such as heme oxygenase (HO) and cyclooxygenase-2 (COX-2), as well as different micro ribonucleic acids (miRNAs) that could play a role in the control of expression of these factors.

## 2. Search Methodology and Data Analysis

The research of articles was carried out on the main scientific databases PubMed, Scopus, and Web of Science. The following search keywords were used: *larynx* OR *laryngeal* AND *cancer* OR *carcinoma* AND *heat shock proteins* OR *HSP* OR *metallothioneins* OR *MT* OR *nuclear factor erythroid 2-related factor 2* OR *Nrf2* OR *heme oxygenase* OR *HO* OR *cyclooxygenase-2* OR *COX-2* OR *micro ribonucleic acid* OR *miRNAs*. Some manuscripts detected in the reference sections of selected articles were also analyzed. Inclusion criteria were prospective and retrospective original articles written in the English language, which provided data about the expression levels of the biomarkers and the possible correlation with LSCC, studies about at least one of such biomarkers as HSPs, MTs, Nrf2, HO, COX-2, and/or miRNAs, studies referring LSCC, and both in vitro studies and human clinical studies. Actually, some studies were carried out in the laboratory using a Hep-2 cell line that, although considered to be derived from an epidermoid carcinoma of the larynx, was established via HeLa cell contamination, as reported by the American Type Culture Collection (ATCC) organization (https://www.atcc.org/products/ccl-23, accessed on 9 October 2023).

Exclusion criteria were review, editorials, case reports, studies that referred only to benign laryngeal lesions, studies that referred overall to head and neck cancers, studies referring to non-squamous cell carcinomas of the larynx, and research of the biomarker in the serum.

**Figure 1:**
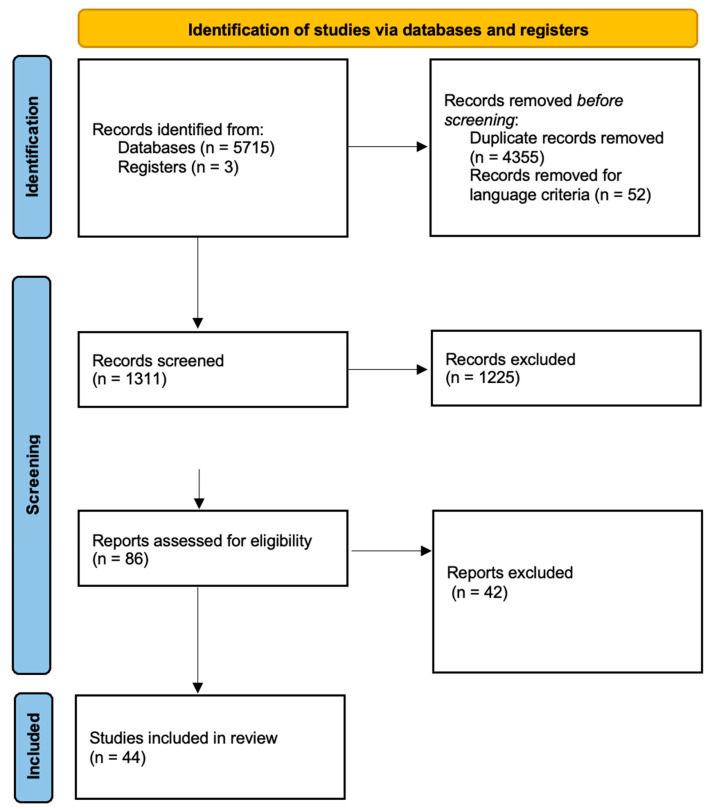
Prisma 2020 flow diagram for review [20].

From the systematic research on databases and references, 5718 manuscripts were selected. Before the screening, 4355 duplicates and 52 articles not written in English were excluded. Then, two authors (B.V. and C.S.) analyzed 1311 manuscripts and excluded 1225 of them via reading title and abstract. So, 86 articles were read in their entirety, and based on the selection criteria, 44 manuscripts were included in this review: 5 articles about HSPs; 4 studies about MTs; 3 articles about Nrf2; 3 articles about HO; 13 manuscripts about COX-2; and 16 studies about miRNAs (Figure 1) [20].

## 3. Biomarkers

### 3.1. Heat Shock Proteins (HSPs)

HSPs are stress-induced proteins involved in intracellular protection mechanisms. The main stress factors responsible for HSP induction are elevated temperature, oxidative stress, hypoxia, heavy metals, ethanol, infections, and radiation. Chaperone and HSP are usually used as synonyms. They are classified according to their molecular weight (expressed in kiloDaltons). They have canonical functions pertinent to maintaining protein homeostasis; indeed, they help the correct folding of many proteins and protect cells from protein misfolding, premature degradation, or aggregation [21]. They also have non-canonical functions, such as participation in immune system regulation, cell differentiation, and carcinogenesis [22,23,24]. Typically, they are cytoprotective, but if qualitatively and/or quantitatively abnormal, they can become pathogenetic and cause a disease called chaperonopathy. Studies demonstrated that some malignant tumors can be classified as “*chaperonopathies by mistake*” since HSPs contribute to cancer cell proliferation and their resistance against antitumor mechanisms [25]. So, in this case, HSPs help tumor survival rather than protecting the host.

Studies found that their overexpression and their role in carcinogenesis depend on the type of cancer [26,27]. For instance, HSP27 overexpression in breast, liver, and prostate cancers is related to poor prognosis, whereas its high expression in esophageal and lung squamous cell carcinoma is related to good prognosis [26,28,29].

A few studies about HSP expression in LSCC investigated only three proteins of this family: HSP27; HSP70; and HSP47 (Table 1).

#### 3.1.1. HSP27

HSP27 represents a small heat shock protein (sHSP) whose effects depend on its phosphorylation, oligomerization, and possible chimera formation with other sHSP. HSP27 usually inhibits apoptosis and necrosis by blocking the caspase system, and it is involved in nuclear protein folding regulation and cell differentiation, too [21]. This chaperone is overexpressed in different types of tumors (breast, lung, prostate, head, and neck) and plays a role in carcinogenesis by promoting anti-apoptotic activity [21,24,30]. In cancer, phosphorylation changes the affinity of HSP27 for its oncoprotein, leading to the activation of anti-apoptotic and pro-survival signaling pathways. This promotes tumor growth, metastatization, and chemoresistance.

**Table 1:** Included studies about heat shock proteins’ (HSP) role in laryngeal squamous cell carcinoma.

Authors	Lee [28]	Karam [29]	Kaigorodova [31]	Xu [32]	Song [26]
Year of publication	2007	2017	2016	2010	2017
Study design	Prospective	Prospective Case–control study	Retrospective	Prospective	Retrospective
Sample size	-	44	50	50	62
HSP	27	27	27	70	47
Expression level of HSP	Upregulation	Upregulation	Upregulation	Upregulation	Upregulation
Correlation between expression levels and LSCC	Cisplatin resistance	Advanced stage Poor differentiated	Neck node metastases	Advanced stage RT resistance	Inhibition of cancer cell proliferation Induction of apoptosis Increased sensitivity to cisplatin

HSP: heat shock proteins; LSCC: laryngeal squamous cell carcinoma; RT: radiotherapy.

In the case of LSCC, HSP27 is found both in the cytoplasm and in the nucleus. In particular, a study carried out on 50 samples of LSCC reported a correlation between the cytoplasmatic expression of phosphorylated HSP27 and nuclear expression of both phosphorylated and non-phosphorylated HSP27 and neck node metastases [31]. Moreover, the expression level of HSP27 is found to be related to tumor stage (*p*-value 0.0039) and grade of differentiation (*p*-value < 0.001) [29]. A 2007 study found that overexpression of this protein in LSCC induces chemoresistance against cisplatin without effects against RT. Indeed, HSP27 seems to arrest cells at the G1 phase, conferring resistance to cisplatin, which primarily acts in the S phase [28]. Therefore, the research on HSP27 in cancer cells could be useful in the case of advanced LSCC, where induction chemotherapy could be proposed.

An in vitro study demonstrated that overexpression of HSP27 in LSCC cells showed two-fold higher survival than cells without biomarker expression after cisplatin exposition since it seems to delay cell proliferation [28]. Indeed, in this study, the authors pretreated control LSCC cells (without HSP27) with mimosine, which arrests cells in the G1 phase, mimicking the role of HSP27. Then, these cells were exposed to cisplatin, finding higher survival than LSCC cells without mimosine and demonstrating the HSP27 mechanism of action in chemoresistance.

#### 3.1.2. HSP70

HPS70 is involved in the control of cellular homeostasis. It plays a role in inhibiting apoptosis induced by reactive oxygen species (ROS). HSP70 overexpression is related to poor prognosis in endometrial and breast cancers [27]. Xu et al. suggested that the expression level of HSP70 was higher in the advanced stage than in the early stage of LSCC (*p*-value 0.015) [32]. In particular, overexpression of HSP70 induces radiotherapy (RT) resistance by inhibiting ROS-induced apoptosis promoted by X-ray. In particular, HSP70 inhibits the degradation of C23, a nuclear protein involved in cell proliferation promotion, induced by RT through ROS.

#### 3.1.3. HSP47

HSP47, also called colligin-2, is a collagen protein of the endoplasmic reticulum involved in the production of procollagens. It is overexpressed and related to carcinogenesis and poor prognosis in several tumors (oral cavity, breast) but not in all types of cancers [33].

**Figure 2:**
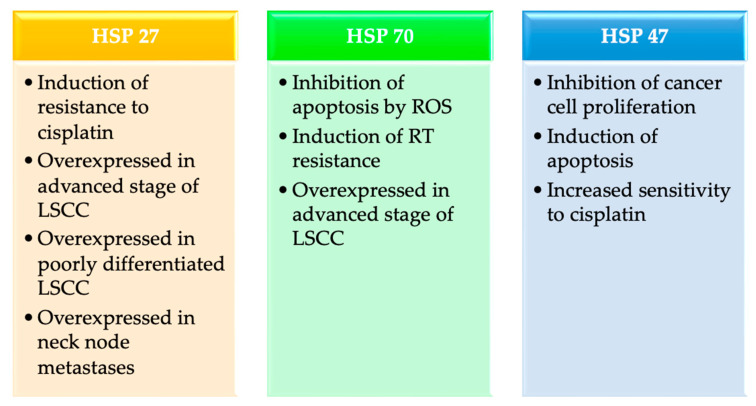
Heat shock proteins’ (HSP) role in laryngeal squamous cell carcinoma (LSCC) [ROS: reactive oxygen species; RT: radiation therapy].

However, in the larynx, HSP47 expression is higher in normal tissue than in LSCC, and it decreases with a decrease in the degree of differentiation of cancer (*p*-value < 0.001). A study showed that this chaperone inhibits cancer cell proliferation, induces apoptosis (via intrinsic and extrinsic pathways), and increases sensitivity to cisplatin. So, in this case, its high expression is related to better prognosis and longer OS (*p*-value 0.001). In vitro study confirmed the following result: overexpression of HSP47 inhibited cancer cell proliferation [26] (Figure 2).

### 3.2. Metallothioneins (MTs)

Metallothioneins are cysteine-rich proteins that bind metals (copper and zinc), protecting cells against ROS, radiation, and heavy metal toxicity. In fact, thanks to the thiol group of their cysteine, MT can bind heavy metals (cadmium, platinum, mercury), protecting cells from their toxicity, whereas, in physiological conditions, MTs bind zinc and copper, promoting cell proliferation. MT1 and MT2 are the most expressed among the 11 human MT isoforms. By the way, MT1 and MT2 expression is induced by different stimuli such as metals, oxidants, and stress. Studies have hypothesized the mechanisms of action of MTs in cancer progression. In particular, the binding of zinc by the MTs can lead to the following possible effects: (1) zinc promotes G1/S phase transition; therefore, low MT levels may arrest cancer cells in the G1 phase, inhibiting their proliferation, and (2) zinc is fundamental for the transcription of tumor suppressor protein as p53; therefore, high MT levels may remove zinc from p53, causing its inhibition and promoting uncontrolled cancer cell growth [34].

Also, in this case, MT expression depends on the type of cancer: upregulated in breast and nasopharyngeal tumors and downregulated in prostate and thyroid carcinoma. Moreover, their overexpression is related to poor prognosis in breast cancer and good prognosis in rectal carcinoma. In the case of lung cancer, MTs are expressed in squamous cell carcinoma, but they are not encountered in small cell cancer [34].

Studies demonstrated their higher expression in malignant lesions than in dysplasia or benign lesions of the larynx (*p*-value < 0.0001 [35]; *p*-value 0.0004 [36]). However, for LSCC, no statistically significant differences were found in MT expression level between different grades of tumor differentiation [35,37]. So, these results demonstrated that MTs may be useful only to distinguish between benign and malignant laryngeal lesions, considering them as an early biomarker of LSCC. Moreover, a 2014 study analyzed the possible correlation between genetic polymorphism of MT2A and the risk of LSCC. In particular, this study found that the single-nucleotide polymorphism at loci 5 A/G (rs28366003) of the MT2A gene was associated with a high risk (1.6-fold higher) of developing an LSCC (*p*-value < 0.001) [38] (Table 2).

### 3.3. Nuclear Factor Erythroid 2-Related Factor 2 (Nrf2)

Nrf2 is a transcription factor that plays a role in cellular protection against oxidative stress, one of the main causes of DNA damage and carcinogenesis [39]. Under physiological conditions, Kelch-like ECH-associated protein 1 (KEAP1) keeps low Nrf2 expression, whereas, in case of oxidative stress conditions, KEAP1 is inhibited by ROS, and Nrf2 is overexpressed and reaches the nucleus where it activates its corresponding genes encoding cytoprotective enzymes such as heme oxygenase-1 (HO1) and NAD(P)H quinone oxidoreductase 1 (NQO1). Therefore, genetic changes, e.g., gain of function or amplification of Nrf2 or loss of functions or deletion of KEAP1, could lead to carcinogenesis and resistance to RT [40,41]. Its overexpression is related to poor prognosis in lung and esophageal squamous cell carcinoma [42,43].

In the case of LSCC, changes in its oxidative stress pathway seem to be related to resistance to RT (*p*-value 0.03) [44]. Li et al. reported high expression of Nrf2 in advanced LSCC compared to adjacent health tissue (*p*-value < 0.01) regardless of age, TNM stage, and tumor size [45]. Zhou et al. reported higher expression of Nrf2 in cancer cell nuclei than in normal surrounding cells and found that its overexpression is related to cisplatin resistance of LSCC. Actually, ROS induced by cisplatin led to the activation of intranuclear Nrf2, which reduces cancer response to chemotherapy [46] (Table 3).

### 3.4. Heme Oxygenase (HO)

Heme oxygenase-1 (HO-1) is an enzyme that protects cells (including cancer cells) against oxidative stress, inflammation, and apoptotic effect [47]. HO-1 is a target of Nrf2. Under cellular oxidative stress conditions, Nrf is activated and migrates into the cell nucleus, where it binds to the antioxidant response element (ARE) area of the HO-1 gene. Studies found that HO-1 is usually more expressed in cancer tissue (e.g., squamous cell carcinoma, lymphosarcoma, melanoma) than in close healthy tissues [48].

Lv et al. carried out a laboratory study on human LSCC Hep-2 cell lines to assess the role of HO-1 in reducing the pro-apoptotic action of cisplatin, one of the most used chemotherapeutics [49]. Cisplatin promotes cancer cell apoptosis by breaking DNA, inhibiting DNA synthesis, or inducing oxidative stress, leading to Nrf2 activation. In their study, the authors found that a high dose of cisplatin is needed to overcome the anti-apoptotic effect of HO-1; however, a high dose of chemotherapeutic means high toxicity and several side effects, too. So, being aware of the HO-1 mechanism of action in cancer, the authors suggest suppressing the enzyme expression to enhance LSCC chemosensitivity to cisplatin. This may lead to a lower cisplatin therapeutic dose and a lower risk of side effects. In their prospective study on 142 patients, Tang et al. found lower HO-1 levels in the LSCC group compared to control ones (*p*-value < 0.001) and that HO-1 levels are inversely correlated with advanced tumor stages (*p*-value < 0.019) and lymph node metastases (*p*-value < 0.001) [50]. On the contrary, the study of Li et al. demonstrated higher HO-1 expression levels in the advanced stage of LSCC than in control samples [45] (Table 4).

### 3.5. Cyclooxygenase-2 (COX-2)

There are two isoforms of cyclooxygenase, COX-1, a constitutive enzyme, and COX-2, which is induced by stress factors (e.g., smoking, RT). COX-2 is an enzyme that catalyzes the production of prostaglandin E2 (PGE2) from arachidonic acid [51]. This enzyme is usually expressed at low levels in healthy tissue; its overexpression is due to inflammatory cytokines and oncogenes. High levels of COX-2 lead to increased production of PGEs that promote carcinogenesis, mainly by angiogenesis, but also by suppressing apoptosis and immune response [52]. This explains why it was found in some tumors such as breast, liver, endometrial, and laryngeal cancers and related to poor prognosis and high risk of recurrence [53,54,55,56]. Sayar et al. compared COX-2 expression levels between LSCC, atypical hyperplasia, and vocal fold nodules. The authors did not find COX-2 staining in vocal fold nodules, whereas there was staining in atypical hyperplasia (45.7% of cases) and LSCC (91.4% of cases). The COX-2 staining intensity was highest in the laryngeal cancer group; however, in this group, a statistically significant relation between COX-2 staining levels and duration of smoking (*p*-value 0.89) and age (*p*-value 0.08) was not found [55].

Ranelletti et al. studied COX-2 expression in LSCC by immunohistochemistry. They found COX-2 immunostaining in well-differentiated laryngeal cancer cells (G1), whereas the poorly differentiated laryngeal cancer cells and the health cells close to the tumor did not express COX-2. By the way, they did not observe a statistically significant correlation between COX-2 expression and TNM stage (*p*-value 0.96), tumor site (*p*-value 0.17), and age (*p*-value 0.78) [57]. Similarly, other studies reported that epithelial immunohistochemical expression of COX-2 is mostly comparable between well-differentiated (G1) and moderate-differentiated (G2) cancer cells, whereas the enzyme is not found in poorly differentiated (G3) cancer cells (*p*-value 0.04) [58,59]. On the contrary, a study conducted on 80 patients affected by LSCC reported a relationship between COX-2 upregulation and advanced stages and poorly differentiated LSCC, correlating high expression levels of this marker with unfavorable prognosis [60]. A 2006 case–control study on 62 samples found higher expression levels of COX-2 in LSCC than in laryngeal normal tissue (*p*-value < 0.01) [61]. Moreover, Kourelis et al. also observed an inverse statistically significant relationship between levels of COX-2 and risk of LSCC recurrence and disease-free survival (DFS) (*p*-value 0.03) [58,62]. Similar results were achieved by Pérez-Ruiz et al., who found a correlation—although not statistically significant—between expression levels of COX-2 and DFS and overall survival [63]. Also, Cho et al. found a statistically significant correlation between COX-2 upregulation and the risk of local relapse in the case of T1-T2N0 LSCC [64]. Then, the authors focused on the microenvironment of the carcinoma, demonstrating that COX-2 expression in cancer-associated fibroblasts (CAFs) could explain the easier tendency to cancer spread and progression through the stroma in poorly differentiated tumors.

A 2022 meta-analysis found that COX-2 overexpression is related to a higher risk of developing LSCC. The authors also reported a correlation between COX-2 expression and T-stage and lymph node metastases; however, this result was found only in the Asian but not the Caucasian population. In conclusion, this meta-analysis demonstrates a statistically significant association between COX-2 overexpression and worse prognosis in LSCC (*p*-value < 0.05) [65]. Sackett et al. reported that upregulation of COX-2 in glottic cancer is related to a higher risk of overall mortality (*p*-value 0.04) and a higher risk of developing a second primary tumor. However, they did not find any statistical correlation between COX-2 overexpression and gender (*p*-value 0.046), smoking habit (*p*-value 0.20), alcohol intake (*p*-value 0.46), TNM stage (*p*-value 0.19) and histology (*p*-value 0.06), or risk of recurrence or mortality related to glottic cancer [66].

COX-2 role in LSCC sensitivity to radiation therapy (RT) was studied by Nix et al. [67]. They demonstrated that laryngeal cancer cells with higher levels of COX-2 are resistant to RT (*p*-value 0.004). So, they hypothesized that assessing the expression levels of this enzyme in pre-treatment LSCC could be a useful prognostic tool: its upregulation may predict the risk of RT failure, recommending surgery as first-line treatment in this case. Moreover, they assumed that selective COX-2 inhibitors, such as NS-398 and SC-236, may enhance sensitivity to RT in cancer cells with COX-2 overexpression [68,69]. By the way, studies demonstrated that inhibition of COX-2 is related to the proliferation of immune suppressor cells, such as the natural killer T (NKT) cells. In fact, in their study, Klatka et al. found that natural killer T (NKT) cell proliferation was lower in the laryngeal cancer group than in the control group (*p*-value < 0.0001). The authors also reported higher expression of NKT cells in the laryngeal cancer group treated with COX-2 inhibitor than in the laryngeal cancer group without this inhibition (*p*-value < 0.0001). Moreover, the NKT cell expression level was higher in the case of early LSCC, well-differentiated tumor (G1), and without neck node metastases (*p*-value < 0.05). So, based on these data, the authors have concluded that a COX-2 inhibitor, called celecoxib, promotes the proliferation of natural killer T (NKT) cells that enhance specific immune responses and suppress tumor angiogenesis, suggesting a possible role of celecoxib in enhancing immunotherapy effect against LSCC [70].

**Figure 3:**
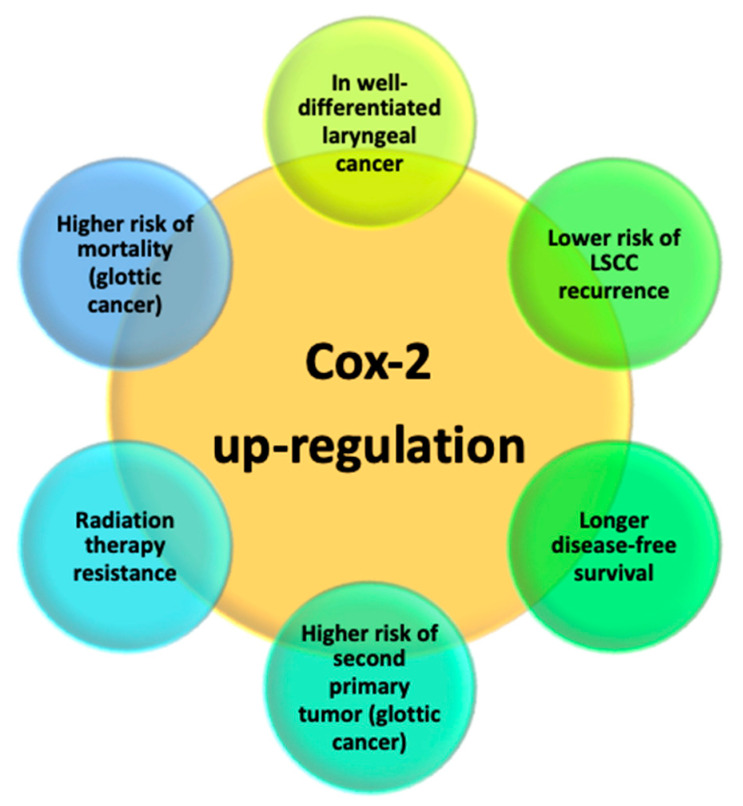
Cyclooxygenase-2 (COX-2) upregulation in laryngeal squamous cell carcinoma (LSCC).

In vitro study on human LSCC Hep-2 found that small hairpin RNA (shRNA)-induced downregulation of COX-2 can inhibit cancer proliferation and invasion and induce cancer cell apoptosis by promoting Caspase-3 activity [71]. This downregulation seems to enhance the sensitivity to Taxanes, which are chemotherapeutic drugs (Table 5) (Figure 3).

### 3.6. Micro Ribonucleic Acids (miRNAs)

MiRNAs are endogenous and non-coding RNAs, usually containing from 19 to 25 nucleotides, that silence target transcripts and impact gene expression. They can have tumor-suppressive or protooncogenic effects, depending on the type of cancer [72]. Epigenetic alterations and defects in enzymes involved in miRNA maturation cause miRNA dysregulation, leading to carcinogenesis. The identification of different expression profiles of miRNAs in the neoplastic tissue compared with normal tissue supports the hypothesis of a probable involvement of miRNAs in tumor development and progression. So, considering the important role of miRNAs in the control of protein expression, the detection of the protein pattern under the control of miRNAs could give important information about specific biomarkers of laryngeal carcinomas.

Studies demonstrate that tobacco smoke may cause changes in the expression level of many miRNAs and cause disrupting mechanisms that are regulated by miRNAs. The expression of miRNAs was found to be distinctive of tumor type, suggesting a role of miRNAs in human carcinogenesis and indicating that miRNAs could be used for tumor classification, diagnosis, and prognosis. For instance, Hs_miR-183_5p is upregulated in liver and colorectal cancers, whereas it is downregulated in breast cancer [73,74].

A recent study compared the expression of nine miRNAs in benign, premalignant, and malignant laryngeal lesions and found a statistically significant expression of *Hs_miR-21_5p, Hs_miR-218_3p,* and *Hs_miR-210_3p* only in the malignant laryngeal lesions. This result could suggest miRNA’s role as a biomarker for laryngeal cancers [75,76,77,78]. In particular, Kinoshita et al. explained the role of *Hs_miR-218_3p* in the regulation of migration and invasion of tumor cells via local adhesion pathways: these data could clarify the mechanism of local recurrence and distant metastasis [79]. Also, Hs_*miR-744-3* upregulation seems to be related to neck node metastasis [80], while *Hs_miR-138* seems to be negatively correlated to distal metastases in LSCC [81]. Tuncturk et al. also found that *Hs_miR-183_5p, Hs_miR-155_5p,* and *Hs_miR-106b_3p* expressions resulted in being statistically significant in both premalignant and malignant lesions, suggesting their role as transformation biomarkers maybe helping to determine the malignancy potential of laryngeal lesions [75]. In particular, *Hs_miR-183_5p* overexpression is positively correlated to neck node metastasis in supraglottic cancer [75]. Meanwhile, *Hs_miR-375* seems to be downregulated in supraglottic cancer (*p*-value 0.037) and in the case of alcohol abuse (*p*-value < 0.05) [76].

As written above, cigarette smoking is one of the main risk factors for laryngeal cancer. So, many studies focused on its effect on miRNA changes and deregulation, leading to impairment of the p53 pathway, too [82,83]. Based on these assumptions, a study correlated smoke habits with miRNA changes in laryngeal cancer and found that *Hs_miR-202* is overexpressed in smokers for more than 20 years (*p*-value 0.005); however, its expression level is not found to be related to LSCC stage (*p*-value 0.087). *Hs_miR-548* is downregulated in smokers for more than 20 years (*p*-value 0.004), but its expression level statistically increases in pT4N+ LSCC (*p*-value 0.030). This study also demonstrated that the *Hs_miR-29a* expression level is statistically significantly lower in T1 than in T2 or T3 LSCC (*p*-value 0.037), without differences based on smoking habit duration (*p*-value 0.096). This result confirms that *Hs_miR-29a* expression is positively correlated with the TNM stage, suggesting its role as an oncogene in laryngeal cancer. The authors also found that *Hs_miR-4768-3p* is negatively correlated with neck node metastasis (*p*-value 0.018). Moreover, it is downregulated in smokers for less than 20 years (*p*-value 0.036). These data suggest its role as a tumor suppressor in LSCC [84]. Expression of *miR-149b* in LSCC was found to be related to histological grade and TNM and 5-year overall survival (*p*-value <0.05), acting as an oncogene [85].

Recent in vitro studies on human LSCC Hep-2 cell lines found that *miR-33a*, *miR-199a-5p*, *miR-145, miR-34a,* and *miR-150-5p* overexpression promoted cancer cell apoptosis, suggesting a role as tumor suppressor [86,87,88,89,90]. A study carried out on 97 patients with LSCC demonstrated that low expression levels of *miR-196b* are related to worse overall survival (median survival: 48 months), while its overexpression is related to better survival (median survival: 81 months) (*p*-value 0.04) [91].

Moreover, a 2019 study reported low expression levels of *miR-143-3p* in 52 LSCC samples, relating this value to the advanced stage and poorly differentiated tumor. In vitro analysis also showed that its overexpression induced cancer cell apoptosis and inhibited cell proliferation and migration [92]. Maia et al. enrolled 34 LSCC samples and found that *miR-296-5p* overexpression was related to resistance to RT (*p*-value 0.010) and a higher risk of recurrence in the early stage (*p*-value 0.025) [93] (Table 6 and Table 7).

## 4. Conclusions

Despite the increased awareness of the carcinogenic risks related to smoking and alcohol habits and the now customary use of more sensitive diagnostic techniques, laryngeal cancer represents the second most common carcinoma among head and neck cancers, with a survival rate below 50% in advanced stages. Because of these data and poor prognosis, recent studies focus on the identification of biomarkers that may play a critical role in the pathogenesis of laryngeal squamous cell carcinoma or that may correlate with any factors (e.g., age, tumor stage, risk factors). However, this review showed that the study of biomarkers in LSCC is still at the beginning, and further research is needed to achieve the main goal: the detection of a therapeutic strategy tailored for each patient in order to ensure the greatest success rate.

## 5. Future Directions

The study of biomarker expression in LSCC and the detection of a possible correlation with patients’ epidemiological, clinicopathological, and therapeutics data may lead to better knowledge of LSCC, the identification of the best therapeutic strategy, and the most proper follow-up protocol tailored for each patient, improving the prognosis of LSCC patients.

## Figures and Tables

**Table 2:** Included studies about metallothioneins’ (MTs) role in laryngeal squamous cell carcinoma.

Authors	Ioachim [35]	Nowinska [36]	Pastuszewski [37]	Starska [38]
Year of publication	1999	2016	2007	2014
Study design	Prospective	Prospective	Retrospective	Prospective
Sample size	73	83	65	323
MTs	MT	MT-I/II	MT	Loci—5 A/G (rs28366003) of the MT2A gene
Expression level of MTs	Upregulation	Upregulation	Upregulation	Genetic polymorphism
Correlation between expression levels and LSCC	Predictive of malignant transformation of laryngeal lesion	Higher in LSCC than in benign laryngeal lesions	Predictive of malignant transformation of laryngeal lesion	High risk of developing LSCC

MTs: metallothioneins; LSCC: laryngeal squamous cell carcinoma.

**Table 3:** Included studies about Nrf2′s role in laryngeal squamous cell carcinoma.

Authors	Sheth [44]	Li [45]	Zhou [46]
Year of publication	2021	2016	2019
Study design	Retrospective	Prospective	Prospective
Sample size	20	33	32
Marker	Nrf2	Nrf2	Nrf2
Expression level of Nrf2	Changes in Nfr2/KEAP1 oxidative stress pathway	Upregulation	Upregulation
Correlation between expression levels and LSCC	RT resistance	Advanced stage	Cisplatin resistance

Nrf2: nuclear factor erythroid 2-related factor 2; LSCC: laryngeal squamous cell carcinoma; RT: radiotherapy.

**Table 4:** Included studies about HO’s role in laryngeal squamous cell carcinoma.

Authors	Lv X [49]	Tang [50]	Li [45]
Year of publication	2016	2016	2016
Study design	Prospective	Prospective Case–control study	Prospective
Sample size	-	142	33
Marker	HO-1	HO-1	HO-1
Expression level of HO	Inhibition	Downregulation	Upregulation
Correlation between expression levels and LSCC	Increased cisplatin sensitivity	Advanced stage Neck node metastases	Advanced stage

HO: heme oxygenase; LSCC: laryngeal squamous cell carcinoma; RT: radiotherapy.

**Table 5:** Included studies about COX-2′s role in laryngeal squamous cell carcinoma.

Authors	Year of Publication	Study Design	Sample Size	Expression Level of COX-2	Correlation between Expression Levels and LSCC
Sayar [56]	2013	Prospective	105	Upregulation	LSCC and smokers
Ranelletti [57]	2001	Prospective	61	Downregulation	Poorly differentiated LSCC and normal laryngeal tissue
Kourelis [58]	2009	Retrospective	52	Downregulation	Higher risk of local recurrence Shorter DFS
Kawata [59]	2006	Prospective	24	Upregulation	Well-differentiated
Chen [60]	2013	Retrospective	80	Upregulation	Advanced stage Poorly differentiated
Chen [61]	2006	Prospective Case–control study	62	Upregulation	LSCC
Kourelis [62]	2007	Prospective	129	Upregulation	LSCC
Pérez-Ruiz [63]	2012	Retrospective	59	Upregulation	Better OS Longer DFS
Cho [64]	2004	Prospective	123	Upregulation	Higher risk of local relapse in T1-T2N0
Sackett [66]	2008	Prospective	301	Upregulation	Higher risk of second primary tumor Higher overall mortality
Nix [67]	2004		122	Upregulation	Higher resistance to RT
Klatka [70]	2017	Prospective Case–control study	78	Inhibition (through celecoxib)	Higher sensitivity to immunotherapy
Wang [71]	2008	Prospective	-	Inhibition (through shRNA)	Induction of cancer cell apoptosis Inhibition of cancer proliferation and invasion

COX-2: cyclooxygenase 2; LSCC: laryngeal squamous cell carcinoma; RT: radiation therapy; DFS: disease-free survival; shRNA: small hairpin RNA; OS: overall survival.

**Table 6:** Included studies about miRNAs’ role in laryngeal squamous cell carcinoma.

Authors	Year of Publication	Study Design	Sample Size	miRNAs	Expression Level of miRNAs	Correlation between Expression Levels and LSCC
Tuncturk [75]	2021	Prospective	30	miR-183_5p	Upregulation	Neck node metastasis in supraglottic cancer
				miR-155_5p	Upregulation Upregulation	Both in premalignant and malignant lesions
				miR-106b_3p		
Bruzgielewicz [84]	2017	Prospective	48	miR-29a	Upregulation	Early stage
				miR-202	Upregulation	Advanced stage Long-time smokers
				miR-4768-3p	Downregulation	Advanced stage with nodal metastases
				miR-548	Upregulation	Advanced stage with nodal metastases
Karatas [86]	2018	Prospective	-	miR-33a	Upregulation	Cancer cell apoptosis
Li [87]	2020	Prospective	25	miR-199a-5p	Upregulation	Cancer cell apoptosis
Hu [76]	2014	Prospective Case–control study	46	miR-21	Upregulation	Biomarker for laryngeal cancers Worse prognosis
				miR-375	Downregulation	Supraglottic cancer Alcohol abuse
Gao [81]	2015	Prospective Case–control study	30	miR-138	Downregulation	Distal metastases Worse prognosis
Erkul [77]	2017	Retrospective Case–control study	72	miR-21	Upregulation	Biomarker for laryngeal cancers
Karatas [88]	2016	Prospective Case–control study	80	miR-145	Downregulation	Tumor suppressor
Shen [89]	2012	Prospective Case–control study	69	miR-34a	Downregulation	Shorter DFS Tumor suppressor
Xu [85]	2016	Prospective	97	miR-149	Downregulation	Shorter OS
Luo [91]	2018	Prospective	79	miR-196b	Upregulation	Worse prognosis
Chen [90]	2023	Prospective Case–control study	16	miR-150-5p	Downregulation	Tumor suppressor
Li [80]	2016	Prospective	-	miR-744-3p	Upregulation	Neck node metastasis
Zhang [92]	2019	Prospective	52	miR-143-3p	Downregulation	Advanced stage Poorly differentiated Shorter OS
Maia [93]	2015	Retrospective	34	miR-296-5p	Upregulation	Resistance to RT Recurrence in early stage
Liu [78]	2009	Prospective	210	miR-21	Upregulation	Biomarker for laryngeal cancers

miRNAs/miR: micro ribonucleic acid; LSCC: laryngeal squamous cell carcinoma; RT: radiation therapy; DFS: disease-free survival; OS: overall survival.

**Table 7:** Micro ribonucleic acid (miRNA) in laryngeal squamous cell carcinoma.

Micro Ribonucleic Acid (miRNAs)	Overexpression
*Hs_miR-21_5p*	Biomarker for laryngeal cancers: only in the malignant laryngeal lesions
*Hs_miR-210_3p*	
*Hs_miR-183_5p*	Positively related to neck node metastasis in supraglottic cancer
*Hs_miR-155_5p*	“Transformation biomarkers”: both in premalignant and malignant lesions
*Hs_miR-106b_3p*	
*Hs_miR-4768-3p*	Negatively correlated with neck node metastasis Downregulated in smokers for less than 20 years
*Hs_miR-29a*	Positively related to tumor stage
*Hs_miR-202*	Overexpressed in smokers for more than 20 years
*Hs_miR-548*	Overexpressed in advanced laryngeal cancer (T4N+)
*Hs_miR-33a* *Hs_miR-199a-5p* *Hs_miR- 150-5p*	Tumor suppressor Cancer cell apoptosis
*Hs_miR-375*	Downregulated in supraglottic cancer and in alcohol abuse
*Hs_miR-138*	Negative related to distal metastases
*Hs_miR-145*	Tumor suppressor
*Hs_miR-34a*	Positively related to disease-free survival
*Hs_miR-149*	Negative related to prognosis Negative related to prognosis
*Hs_miR-196b*	
*Hs_miR-744-3p*	Neck node metastasis
*Hs_miR-143-3p*	Downregulated advanced and poorly differentiated laryngeal cancer
*Hs_miR-296-5p*	Resistance to radiation therapy Risk of recurrence in early stage

## Data Availability

Publicly available datasets were analyzed in this study. These data can be found here: https://pubmed.ncbi.nlm.nih.gov; https://www.scopus.com; https://www.webofscience.com/wos/author/search; accessed on 20 August 2023.
